# Early- vs. late-onset colon cancer: clinicopathological insights and survival outcomes in an East Asian cohort

**DOI:** 10.1007/s00384-025-05007-4

**Published:** 2025-10-09

**Authors:** Chun-Chi Lin, Che-Yuan Chang, Yu-Zu Lin, Hou-Hsuan Cheng, Sheng-Chieh Huang, Hung-Hsin Lin, Yuan-Tzu Lan, Huann-Sheng Wang, Shih-Ching Chang, Shung-Haur Yang, Jeng-Kai Jiang, Wei-Shone Chen, Hao-Wei Teng, Yi-Wen Yang

**Affiliations:** 1 Division of Colon and Rectal Surgery, Department of Surgery, Taipei Veterans General Hospital, Taipei City, Taiwan; 2 Faculty of Medicine, School of Medicine, National Yang Ming Chiao Tung University, Taipei City, Taiwan; 3 National Yang Ming Chiao Tung University Hospital, Yilan City, Yilan County Taiwan; 4 En Chu Kong Hospital, New Taipei City, Taiwan; 5 Division of Medical Oncology, Department of Oncology, Taipei Veterans General Hospital Taipei, Taipei City, Taiwan

**Keywords:** Colon cancer, Cancer-specific survival, Prognostic factors, Early-onset colon cancer, Age-related survival outcomes

## Abstract

**Purpose:**

Colorectal cancer (CRC) is the third most prevalent cancer worldwide, showing an increasing early-onset CRC in patients, which is defined as diagnoses made before the age of 50. Studies conflict on early-onset CRC prognosis versus late-onset CRC with varying survival outcomes. This study explores the age-related survival differences in colon cancer by comparing early- and late-onset groups.

**Methods:**

We performed a retrospective cohort study at a tertiary referral hospital (2010–2018), including 3459 patients with colon cancer (3076 late-onset, 383 early-onset). The clinicopathological features of early- and late-onset colon cancer were compared, and cancer-specific survival was evaluated using the Kaplan–Meier analysis with log-rank tests. The multivariate Cox regression identified independent prognostic factors.

**Results:**

Early-onset colon cancer showed female predominance, better Eastern Cooperative Oncology Group performance, more left-sided tumors, and advanced stages. On the contrary, late-onset patients had worse cancer-specific survival (hazard ratio 1.506, 95% confidence interval 1.147–1.977, *p* = 0.003), particularly in stages II/III, with tumor perforation, signet ring cells, or no perineural invasion.

**Conclusion:**

In conclusion, despite early-onset colon cancer exhibiting more aggressive features, it is associated with better survival compared with late-onset cases. Further studies are required to validate these findings.

**Supplementary Information:**

The online version contains supplementary material available at 10.1007/s00384-025-05007-4.

## Introduction

Colorectal cancer (CRC) is the third most prevalent cancer and is the second leading cause of cancer-related mortality worldwide [1]. Over 130,000 new CRC cases are identified annually in the United States alone, with approximately 1 million new patients and over 500,000 deaths recorded worldwide each year [2]. Early-onset CRC (EOCRC) refers to CRC diagnosed at < 50 years of age. Despite an overall decline in CRC incidence in the general population, EOCRC incidence is increasing worldwide [3–5]. Although the precise causes of this trend are unclear [6], effective screening programs targeting individuals aged 50–75 at average risk [7, 8], combined with polypectomy, may contribute to a lower-than-expected CRC incidence [9].

Previous studies suggest that EOCRC frequently presents with distinct histological subtypes, including more aggressive ones such as mucinous, signet ring carcinomas, and a poorly differentiated histology [10–14]. It is more commonly located in the distal colon and rectum [12, 15]; it is also associated with a higher occurrence of synchronous and metachronous tumors [16]. Overall, the disease tends to present at more advanced stages in younger individuals [17].

Whether or not young patients with CRC exhibit a distinct biological behavior and carry a different prognosis remains controversial. While some studies showed that patients with EOCRC have a poorer survival compared with older individuals [10, 18–21], some others suggest that although EOCRC patients are more likely to have advanced-stage disease, they also receive a more intensive treatment. Consequently, their overall and stage-specific survival rates appear comparable to or even better than patients with late-onset CRC (LOCRC) [14, 22–27]. These conflicting data could be caused by factors such as the heterogeneity between different studies in terms of patient numbers, treatment (e.g., curative resection or neoadjuvant therapy), and failure to adjust for potential confounding factors.

These factors can substantially influence the research outcomes. This study aims to assess how age at diagnosis affects survival after colon cancer treatment while meticulously adjusting for disease-related variables to highlight the importance of enhancing the risk assessment and screening strategies for younger adults.

We seek to elucidate the impact of age on survival outcomes in patients with colon cancer while also comparing patients with early-onset colon cancer with late-onset ones.

## Methods

This retrospective observational study selected the patients with colon cancer treated at a tertiary referral hospital in Taiwan between 2010 and 2018. The primary endpoint herein was the difference of cancer-specific survival between patients with early- and late-onset colon cancer. We systematically enrolled consecutive patients who were diagnosed with colon adenocarcinoma and underwent surgical resection of the primary tumor. Individual patient medical records were thoroughly reviewed to extract the relevant data encompassing the following: (1) patient demographic characteristics, including sex, age, family history, and levels of tumor markers (e.g., carcinoembryonic antigen [CEA]); (2) tumor characteristics, including location, TNM staging, and key pathological prognostic features (e.g., differentiation, lymphovascular invasion [LVI], perineural invasion [PNI], and mucinous component); and (3) follow-up data, including disease recurrence and patient status at the last follow-up.

To avoid heterogeneity, patients who were diagnosed with pathology other than adenocarcinoma and rectal cancers or those who did not undergo definitive surgery, exemplified by cases where only procedures such as bypass or ostomy were performed, were excluded. In addition, patients with missing key variable values (e.g., age, tumor stage, and survival status) were also excluded from the final analysis. Informed consent was waived. The study was approved by the local ethics committee under No. 2024–05-009CC.

As part of our standard practice, patients in our hospital routinely undergo preoperative computed tomography scans of the abdomen and chest such that we can comprehensively stage their condition. We meticulously reviewed the clinical data that encompassed the clinicopathological features and the preoperative blood tests recorded within 14 days before surgery. The pathological cancer staging was assessed after the surgery according to the American Joint Committee on Cancer TNM classification [28]. We also conducted thorough follow-up assessments: the patients received 3-month-interval visits during the first 2 years, 6-month-interval visits for the subsequent 3 years, and yearly visits, thereafter. When clinical necessity arose, we conducted supplementary investigations, including magnetic resonance imaging, whole-body bone scans, and positron emission tomography scans. The last follow-up day was patient death or until November 2023. The study followed the STROBE reporting guidelines. The “STROBE checklist for cohort studies” is provided in the Supplementary Material.

### Definition

The Eastern Cooperative Oncology Group (ECOG) performance status is a scale used to assess a patient’s level of functioning in terms of their ability to care for themselves, their daily activities, and physical abilities (ambulatory status). The ECOG is a 5-point scale, where 0 means fully active without restriction, and 5 is death. The intermediate points include physical activity restrictions and varying degrees of self-care limitations, with increasing bed or chair confinement as the scale progresses [29]. Right-sided colon cancer is defined as a tumor arising from the cecum to the transverse colon, whereas left-sided colon cancer can be found at the splenic flexure of the colon to the rectosigmoid colon. The family history denoted CRC occurrence within the third degree of familial relationships. The neutrophil–lymphocyte ratio (NLR) is calculated by dividing the neutrophil count by the lymphocyte count. Cancer-specific survival (CSS) is defined as the duration from the diagnosis date until death specifically attributed to colon cancer, excluding deaths due to other causes recorded in the database. The survival data were collected from the hospital medical records and the National Death Registry. Loss to follow-up is defined as the absence of clinical records or survival information for more than 12 months prior to the end of the study period. Patients who were lost to follow-up were censored at the date of the last known contact.

### Statistical analysis

The categorical variables were presented as counts with corresponding percentages. The categorical variable variations were evaluated with the *χ*^2^ test or Fisher’s exact test when the expected cell counts were below 5. A survival analysis was conducted by comparing the Kaplan–Meier survival curves and performing the log-rank test. The potential prognostic factors with a *p* value less than 0.20 in the univariate analysis underwent further evaluation through multivariate Cox regression.

All statistical analyses were performed using SPSS software (version 22.0, IBM Corp., Armonk, NY, USA). The forest plot was generated using the Matplotlib library in Python. The data points are displayed with the horizontal lines representing the confidence intervals (CIs), while the reference lines and the labels are included to emphasize the statistical significance and trends. The statistical significance is defined as a two-tailed *p* value less than 0.05. This was a retrospective cohort study; hence, the study size was defined by the number of patients available in the institutional database during the study period. No formal sample size was calculated.

## Results

### Demographic and clinicopathological profiles

**Fig. 1:**
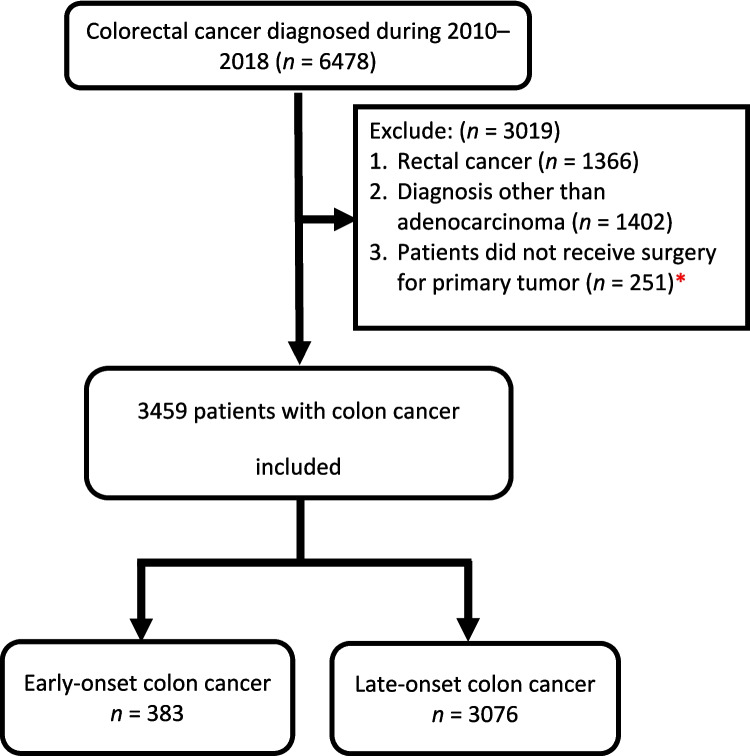
Flowchart of the study population selection. *Among the patients who did not receive surgery for their primary tumor, 98 patients were a clinically stage IV patient. A total of 11 patients had early-onset stage IV colon cancer, whereas 87 had late-onset colon cancer

During this period, 3459 patients with colon cancer were enrolled and analyzed (Fig. 1). The median follow-up was 87.7 months (interquartile range, 72.7–110.4 months) and calculated using the reverse Kaplan–Meier method. Of these, 1494 patients were classified as lost to follow-up; only 137 surviving patients had less than 5 years of follow-up. Table 1 demonstrates the demographic and clinicopathological profiles of the study cohort. The mean age of the cohort was 67.7 years (standard deviation ± 13.6), indicating a predominantly older population. Stratification by age revealed that 88.9% (*n* = 3076) of the participants were aged 50 years or older, whereas only 11.1% (*n* = 383) were younger than 50 years. The tumor location data indicated that 41.5% (*n* = 1436) of the cases were right-sided, whereas 58.5% (*n* = 2023) were left-sided, reflecting a higher prevalence of the left-sided tumors. The staging revealed a distribution across all four stages: stages I (16.7%, *n* = 577), II (33.8%, *n* = 1170), III (30.9%, *n* = 1070), and IV (18.6%, *n* = 642).

**Table 1:** Demographics of the 3459 patients with colon cancer

Characteristic	No	(%)
Age		
Mean ± S.D	67.7 ± 13.6	
Sex		
Male	2010	58.1
Female	1449	41.9
Age		
≧50	3076	88.9
< 50	383	11.1
BMI		
≧25	1166	33.7
< 25	2184	63.1
NA	109	3.2
Family history of CRC		
Yes	560	16.2
No	2899	83.8
ECOG		
0	2597	75.1
1	618	17.9
2	155	4.5
3	33	1.0
4	9	0.3
NA	47	1.4
Location		
Right-sided	1436	41.5
Left-sided	2023	58.5
Stage		
I	577	16.7
II	1170	33.8
III	1070	30.9
IV	642	18.6
Well to moderate	3178	91.9
Poor to undifferentiated	250	7.2
NA	31	0.9
Presence of LVI		
Yes	1071	31.0
No	2379	68.8
NA	9	0.3
Presence of signet ring cells		
Yes	130	3.8
No	3305	95.5
NA	24	0.7
Presence of perineural invasion		
Yes	507	14.7
No	2290	66.2
NA	662	19.1
CEA level (preOP)		
≧5 ng/mL	1336	38.6
< 5 ng/mL	1923	55.6
NA	200	5.8
NLR		
≧3	1104	31.9
< 3	2098	60.7
NA	257	7.4
Oxaliplatin-based adjuvant chemotherapy#		
Yes	673	62.9
No	397	37.1
*BRAF* V600E mutation		
Mutant type	196	5.7
Wild type	2103	60.8
NA	1160	33.5
Microsatellite instability		
MSI-H	128	3.7
MSI-L/MSS	1187	34.3
NA	2144	62.0

*NA*, not available; *LVI*, lymphovascular invasion; *NLR*, neutrophil-to-lymphocyte ratio

^#^The figures and the percentages were calculated for patients with stage III colon cancer

### Descriptive clinicopathological feature analysis by age group

We examined the associations between the clinicopathological characteristics and age dichotomized as ≥ 50 and < 50 years within the current cohort. Table 2 shows that compared with late-onset colon cancer, early-onset colon cancer is significantly female-predominant (*p* = 0.001), has a better ECOG performance status (*p* = 0.001), a more left-sided tumor distribution (*p* = 0.001), has more patients presenting with tumor perforation (*p* = 0.010), has more patients with a family history of CRCs (*p* = 0.001), more frequent poor-to-undifferentiated tumors (0.010), and a stronger presence of the signet ring cells and the PNI (*p* = 0.001 and 0.005, respectively). Among the patients analyzed for the BRAF V600E mutation, those with young-onset colon cancer exhibited a significantly higher mutation prevalence compared with those with late-onset colon cancer (12.1% vs. 8.0%, *p* = 0.022). The stage distribution significantly differed (*p* = 0.001). The late-onset colon cancer group exhibited a higher proportion of stages I (17.5%, *n* = 537 vs. 10.4%, *n* = 40) and II (34.4%, *n* = 1059 vs. 29.0%, *n* = 111) disease, whereas the early-onset colon cancer group showed higher rates of stages III (36.8%, *n* = 141 vs. 30.2%, *n* = 929) and IV (23.8%, *n* = 91 vs. 17.9%, *n* = 551). In other words, there is a tendency toward more advanced and more aggressive diseases in younger patients because they have more tumor perforation, poor-to-undifferentiated tumors, signet ring cells, and PNI. A slightly higher prevalence of the LVI was observed in the patients with early-onset colon cancer, albeit not reaching significance (*p* = 0.065).

**Table 2:** Relationships between various clinicopathological features and age (age ≧50 vs. < 50)

Characteristic	Age				
	≧50	%	< 50	%	*p*
Sex					0.001
Male	1821	59.2	189	49.3	
Female	1255	40.8	194	50.7	
ECOG					0.001
0–1	2840	93.6	375	98.9	
2–4	193	6.4	4	1.1	
BMI					0.054
≧25	1053	35.4	115	30.4	
< 25	1919	64.6	263	69.6	
Location					0.001
Right-sided	1322	43.0	114	29.8	
Left-sided	1754	57.0	269	70.2	
Tumor obstruction					0.125
Yes	712	26.6	103	30.6	
No	1962	73.4	234	69.4	
Tumor perforation					0.010
Yes	63	2.4	16	4.7	
No	2611	97.6	321	95.3	
Family history of CRC					0.001
Yes	467	15.2	93	24.3	
No	2609	84.8	290	75.7	
Stage					0.001
I	537	17.5	40	10.4	
II	1059	34.4	111	29.0	
III	929	30.2	141	36.8	
IV	551	17.9	91	23.8	
Grade of differentiation					0.010
Well to moderate	2839	93.1	339	89.4	
Poor to undifferentiated	210	6.9	40	10.6	
LVI					0.065
Presence	937	30.5	134	35.2	
No	2132	69.5	247	64.8	
Presence of signet ring cells					0.001
Yes	104	3.4	26	6.9	
No	2952	96.6	353	93.1	
Presence of PNI					0.005
Yes	432	17.4	75	23.8	
No	2050	82.6	240	76.2	
CEA level (preOP)					0.737
≧5 ng/mL	1208	41.7	149	40.8	
< 5 ng/mL	1686	58.3	216	59.2	
NLR					0.160
≧3	992	34.8	110	31.1	
< 3	1856	65.2	244	68.9	
Oxaliplatin-based adjuvant chemotherapy#					0.001
Yes	556	59.8	117	83.0	
No	373	40.2	24	17.0	
BRAF V600E mutation					0.022
Mutant type	162	8.0	34	12.1	
Wild type	1856	92.0	247	87.9	
Microsatellite instability					0.120
MSI-H	101	9.2	27	12.6	
MSI-L/MSS	1000	90.8	187	87.4	

^#^The figures and the percentages were calculated for patients with stage III colon cancer

The preoperative CEA levels also showed no significant difference (*p* = 0.737), with 41.7% and 40.8% of the late- and early-onset colon cancer groups, respectively, having levels ≥ 5 ng/mL. Similarly, the NLR did not significantly differ (*p* = 0.160), with 34.8% and 31.1% of the older and younger groups, respectively, having an NLR ≥ 3.

### Descriptive survival analysis in patients with colon cancer

**Fig. 2:**
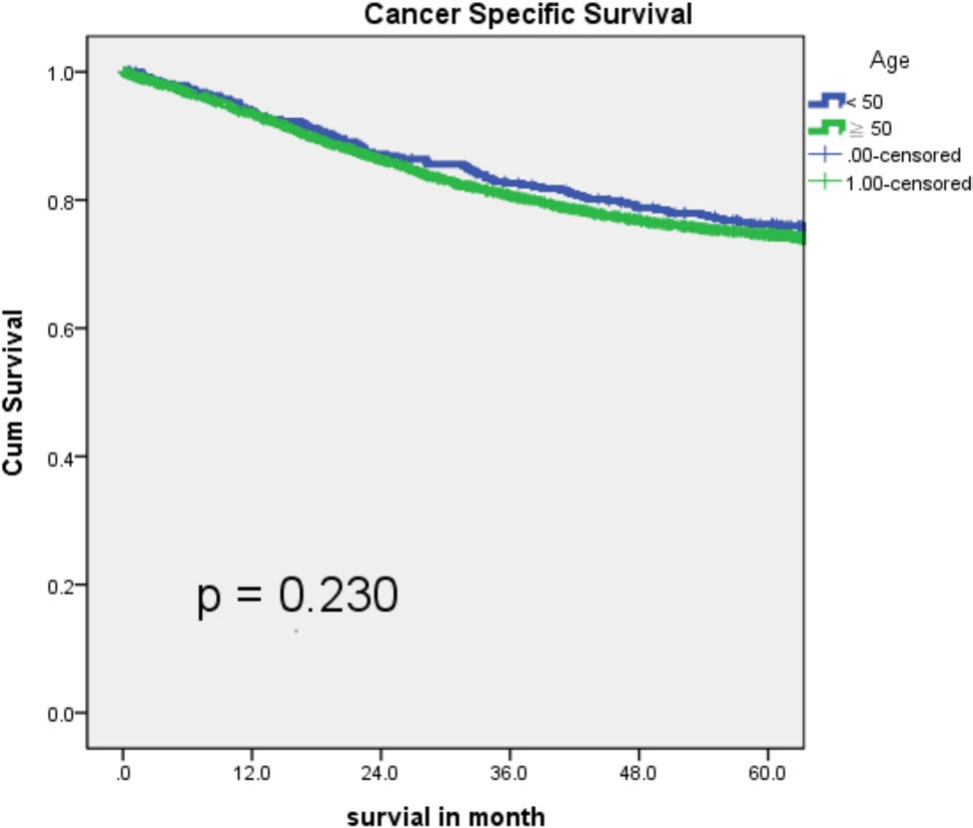

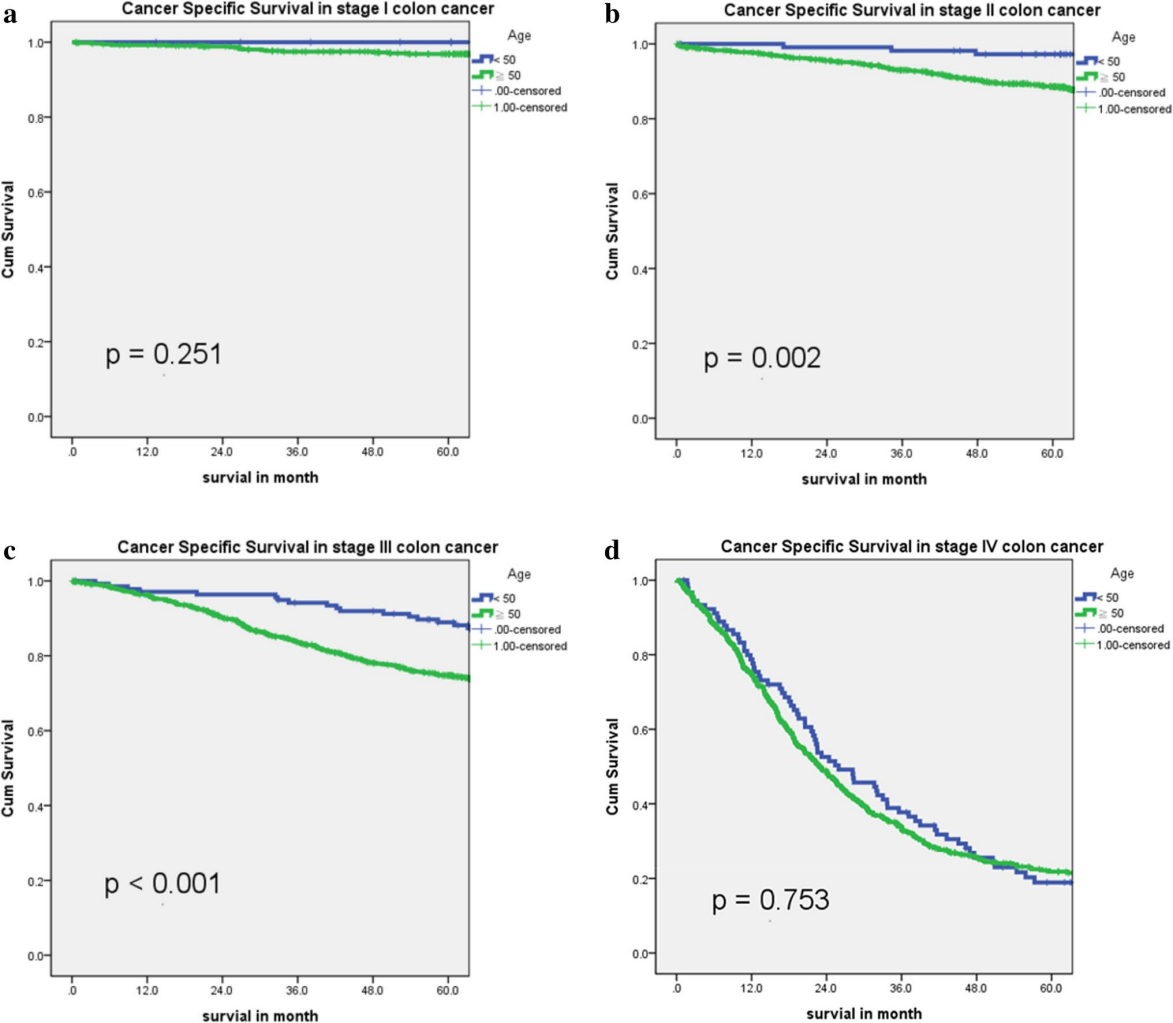
Cancer-specific survival across age categories in the study population. **a** Cancer-specific survival by age group in stage I colon cancer; **b** Cancer-specific survival by age group in stage II colon cancer; **c** Cancer-specific survival by age group in stage III colon cancer; **d** Cancer-specific survival by age group in stage IV colon cancer

The 5-year cancer-specific risk was 23.8% and 25.4% for patients with early- and late-onset cancer, respectively. The univariate analysis identified several potential risk factors for worse CSS in the current cohort (Table 3), including ECOG 2–4, a body mass index (BMI) of less than 25, presence of tumor obstruction or perforation, family history of CRC, advanced staging, poorly to undifferentiation, presence of the LVI, signet ring cells or PNI, elevated preoperative CEA level (≥ 5 ng/mL), and an NLR of more than 3. Meanwhile, the multivariate analysis identified ECOG 2–4, tumor obstruction, tumor perforation, advanced stage (III–IV), poorly to undifferentiation, LVI, PNI, elevated CEA (≥ 5 ng/mL), and NLR ≥ 3 as the prognostic significance for worse CSS after adjustment. Notably, the patients with late-onset colon cancer exhibited significantly worse survival after adjusting for possible tumor-related and clinical confounders (hazard ratio [HR] 1.506, 95% CI 1.147–1.977, *p* = 0.003). Figure 2 displays the Kaplan–Meier plot of the CSS for colon cancer, with Fig. 2a–d illustrating the survival stratified by stages I–IV, respectively. In Supplementary Table 1 and Supplementary Fig. 1, in patients with stage II/III colon cancer, late-onset colon cancer exhibited a significantly worse disease-free survival compared with early-onset colon cancer in the univariate and multivariate analyses (HR 1.827, 95% CI 1.308–2.552, *p* = 0.001 and HR 1.908, 95% CI 1.272–2.862, *p* = 0.002, respectively). In the overall survival analysis, late-onset colon cancer was found to be a significant adverse prognostic factor. Patients with late-onset colon cancer also exhibited significantly worse overall survival compared to those with early-onset colon cancer (HR 1.897, 95% CI 1.475–2.440, *p* = 0.001) (Supplementary Table 2 and Supplementary Fig. 2). We conducted a sensitivity analysis by reanalyzing the data, including only patients with complete follow-up data and excluding the 1494 patients classified as lost to follow-up. The results confirmed a similar survival benefit for early-onset colon cancer, particularly among patients with stages II and III colon cancer (Supplementary Fig. 3a–d).

**Table 3:** Univariate and multivariate analyses for cancer-specific survival in patients with colon cancer

Variable	Univariate analysis			Multivariate analysis		
	HR	95% CI	*p*	HR	95% CI	*p*
Age			0.233			0.003
≧50	1.137	0.921–1.405		1.506	1.147–1.977	
< 50	-	-		-	-	
Sex			0.059			0.797
Male	1.135	0.995–1.295		1.022	0.865–1.208	
Female	-	-		-	-	
ECOG			0.001			0.001
0–1	-	-		-	-	
2–4	2.755	2.220–3.420		1.842	1.307–2.597	
BMI			0.001			0.252
≧25	0.715	0.620–0.825		0.900	0.751–1.078	
< 25	-	-		-	-	
Location			0.070			0.143
Right-sided	1.128	0.990–1.285		1.138	0.957–1.352	
Left-sided	-	-		-		
Tumor obstruction			0.001			0.001
Yes	2.392	2.075–2.757		1.468	1.235–1.744	
No	-	-		-	-	
Tumor perforation			0.001			0.005
Yes	2.588	1.883–3.558		1.881	1.208–2.929	
No	-	-		-	-	
Family history of CRC			0.005			0.772
Yes	0.760	0.628–0.918		0.966	0.766–1.219	
No	-	-		-	-	
Stage						
I	0.016	0.010–0.026	0.001	0.050	0.027–0.930	0.001
II	0.073	0.060–0.088	0.001	0.124	0.094–0.164	0.001
III	0.164	0.141–0.191	0.001	0.296	0.240–0.365	0.001
IV	-	-		-	-	
Grade of differentiation			0.001			0.001
Well to moderate	-	-		-	-	
Poor to undifferentiated	2.013	1.641–2.469		1.643	1.267–2.130	
LVI			0.001			0.001
Presence	3.679	3.229–4.192		1.894	1.563–2.294	
No	-	-		-	-	
Presence of signet ring cells			0.001			0.104
Yes	2.195	1.696–2.841		1.309	0.946–1.810	
No	-	-		-	-	
Presence of PNI			0.001			0.001
Yes	3.327	2.870–3.856		1.578	1.314–1.893	
No	-	-		-	-	
CEA level (preOP)			0.001			0.001
≧5 ng/mL	4.114	3.560–4.756		1.976	1.637–2.386	
< 5 ng/mL	-	-		-	-	
NLR			0.001			0.001
≧3	2.122	1.854–2.429		1.361	1.148–1.614	
< 3	-	-		-	-	
Oxaliplatin-based adjuvant chemotherapy			0.744			0.001
Yes	1.024	0.888–1.180		0.502	0.406–0.619	
No	-	-		-	-

**Fig. 3:**
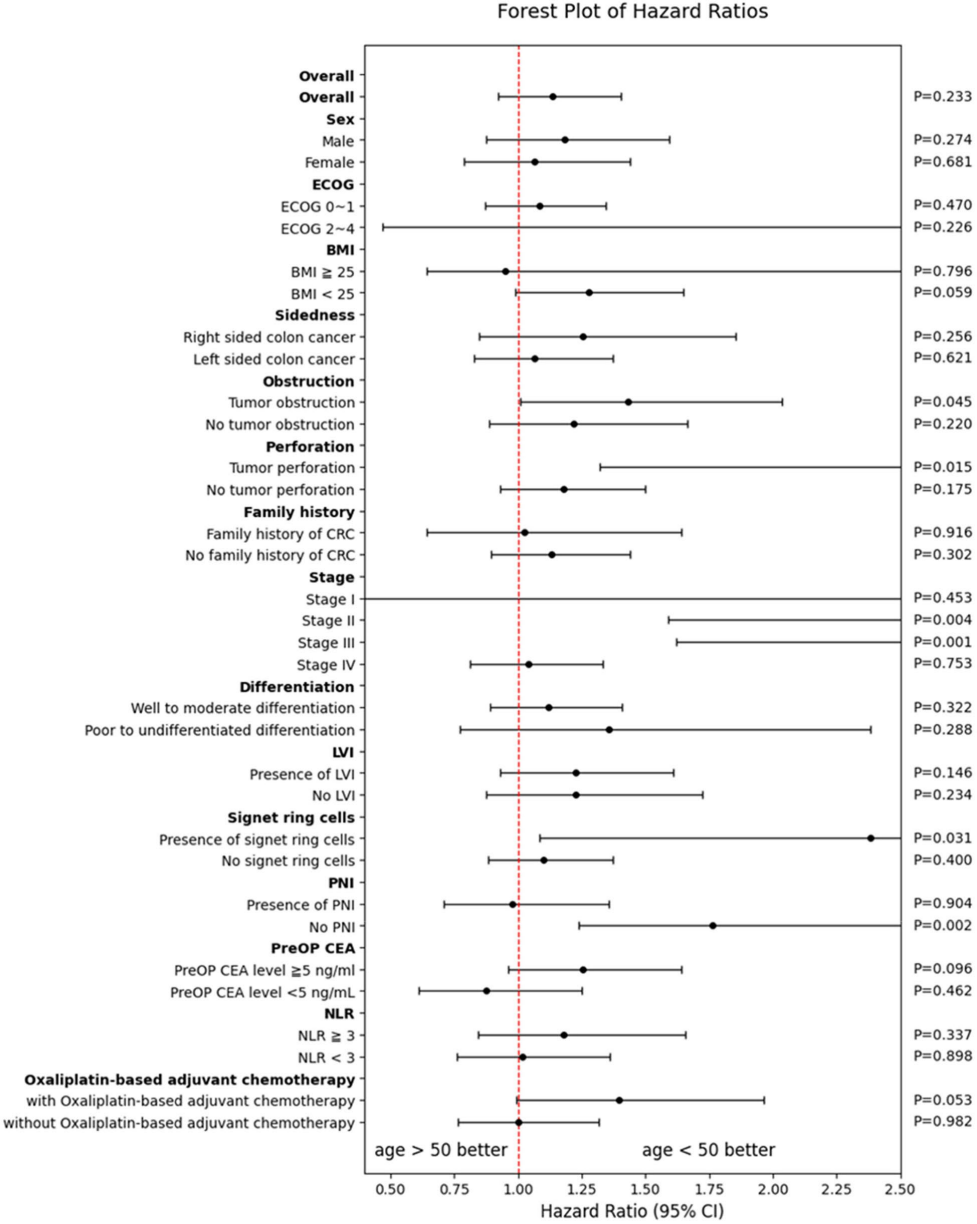
Comparative cancer-specific survival of late- vs. early-onset colon cancer across diverse subgroups

Figure 3 depicts the comparative CSS of late- vs. early-onset colon cancer across the diverse subgroups. Compared with the patients with early-onset colon cancer, the ones with late-onset colon cancer showed significantly worse survival outcomes when presenting with tumor perforation (HR 4.29, 95% CI 1.32–13.95, *p* = 0.015), stages II and III (HR 4.29, 95% CI 1.59–11.60, *p* = 0.004 and HR 2.61, 95% CI 1.62–4.61, *p* = 0.001, respectively), presence of signet ring cells (HR 2.38, 95% CI 1.08–5.23, *p* = 0.031), or absence of the PNI (HR 1.77, 95% CI 1.24–2.51, *p* = 0.002). Sex, ECOG score, BMI, tumor location, and family history of CRC do not show significant survival differences between the age groups.

## Discussion

This study highlights the significant age-related differences in the clinicopathological features within the colon cancer cohort herein. The patients with early-onset colon cancer exhibited a higher prevalence of female sex, left-sided tumors, advanced stages (III and IV), more tumor perforation, more patients with a family history of CRC, and aggressive histopathological features (i.e., poorly differentiation or undifferentiation, presence of signet ring cells, and presence of the PNI). In the univariate analysis of CSS, early-onset colon cancer demonstrated a similar prognosis to late-onset colon cancer. However, after adjusting for multiple clinicopathologic factors, patients with late-onset colon cancer exhibited significantly worse survival compared with those with early-onset colon cancer (*p* = 0.003).

EOCRC is characterized by a lower mutation frequency in certain oncogenes, such as KRAS, BRAF, and NRAS, and a higher mutation prevalence in TP53 and PTEN [30]. Studies also showed that patients with EOCRC exhibit a higher microsatellite instability (MSI)—high status incidence. For instance, one study reported that 10.2% of the EOCRC cases were MSI-high when compared with only 2.2% in the LOCRC cases [31]. Another study found that patients with EOCRC are more likely to have MSI-high tumors with a 26.2% prevalence rate, which is significantly higher than that in older patients [32]. These molecular differences suggest that EOCRC and LOCRC may follow different pathogenic pathways that may influence the prognosis and therapeutic strategies.

In line with the previous publication, EOCRC showed higher rates of poorly differentiated tumors, mucinous adenocarcinoma, and signet ring cell carcinoma [30, 33–35]. In addition, EOCRC and LOCRC significantly differs in their staging at diagnosis. EOCRC is typically diagnosed at more advanced stages compared with LOCRC. A systematic review and a meta-analysis aimed to evaluate the prognosis of early- versus late-onset sporadic colorectal cancer [36]. A total of 26 studies involving more than 1 million patients were analyzed. The findings revealed that a higher percentage of patients with EOCRC (60%) were diagnosed at advanced stages (III–IV) compared with those with LOCRC (49%). This research highlights that EOCRC is often diagnosed at more advanced stages compared with LOCRC, depicting a trend that is consistently observed in this work. Although EOCRC exhibits aggressive characteristics and advanced stages, its survival outcomes remain uncertain compared with those of LOCRC.

Some studies suggested that EOCRC may have worse outcomes. Foppa et al. [37] found that EOCRC had worse recurrence/progression-free survival compared with LOCRC, especially in stage I. However, in recurrence-free survival, deaths without a recurrence are censored, whereas, in progression-free survival, these deaths are categorized as progression events. Combining these two metrics could result in biased survival estimates because it will lead to the inconsistent treatment of patients who die without a recurrence.

Liao et al. [34] recently found that the EOCRC cases exhibit more aggressive disease characteristics, advanced stages at diagnosis, and inferior survival rates compared with LOCRC, thereby emphasizing the need for heightened awareness and early detection, especially among the younger populations. However, the study excluded patients aged more than 70 years; therefore, the study possibly omitted a critical demographic constituting a substantial proportion of the LOCRC cases. This decision artificially narrows the clinical relevance of the findings, particularly given the growing elderly population globally.

Another multicentric retrospective analysis of 1272 metastatic cases published by Pretta et al. [38] revealed that patients with early-onset metastatic CRC tend to have a poorer prognosis compared with those with LOCRC, irrespective of the molecular status. These results emphasized notable disparities that could affect clinical practice and research strategies [38]. In our series, we observed that stage IV early- and late-onset colon cancer exhibited similar survival rates. However, detailed molecular data were not available for further analysis.

A recent systematic review and meta-analysis [36] highlighted the urgent need for increased vigilance in early CRC detection in young patients to reduce the risk of a higher stage at diagnosis. In their analysis, despite being presented with more advanced disease, EOCRC showed a better overall survival compared with LOCRC and equal CSS, disease-free survival, and local and distant recurrence risks. Furthermore, the meta-regression analysis also revealed poorer disease-free survival in the early-onset rectal cancer subgroup, highlighting the need for more uniform and well-designed studies to better elucidate the EOCRC prognosis.

A study analyzing data from the Surveillance, Epidemiology, and End Results database compared the survival outcomes between early- and late-onset metastatic CRC to find the differences and identify the prognostic factors [39]. They concluded that patients with EOCRC had longer survival times compared with those with LOCRC, showing significant differences in the survival outcomes influenced by the tumor location, metastasis, and treatment modalities. Another study using the National Cancer Database showed that after adjusting for stage and other factors, patients with EOCRC exhibited a lower risk of death compared with those diagnosed at ages 51–55 years [40]. The United States Multi-Society Task Force on Colorectal Cancer also noted that despite EOCRC being diagnosed at more advanced stages and having more aggressive histology, the stage-adjusted CSS is better in younger patients compared with those diagnosed over age 50 [4, 5].

Cheng et al. [40] also recently analyzed EOCRC survival by using the National Cancer Database. They found that patients with EOCRC had a lower 10-year survival rate in the unadjusted analysis compared with those diagnosed between ages 51 and 55 (53.6 vs. 54.3%, respectively). However, after adjusting for the factors (e.g., stage), patients with EOCRC had a lower risk of death (adjusted HR 0.95), which is in alignment with our findings.

This current retrospective study conducted at a single institution had several limitations that preclude the broad generalization of its findings owing to potential selection biases, racial factors, and other variables; however, as the cohort primarily comprises East Asian individuals, the results may serve as a reference standard for the East Asian populations. The additional constraints include the unaddressed genetic heterogeneity between EOCRC and LOCRC, which may influence the survival disparities related to age, and the exclusion of patients with rectal cancer owing to diverse treatment modalities, potentially overlooking the critical aspects of CRC management and necessitating further research to clarify the true impact of age on survival outcomes, particularly in patients with rectal cancer, which has remained unaddressed in this study. Among the patients who did not undergo primary tumor resection, 98 were diagnosed with a clinical stage IV disease. The lack of data regarding the reasons for non-surgical management introduces a selection bias potential, necessitating caution in the interpretation of these findings.

## Conclusions

This study revealed distinct age-related clinicopathological differences in a CRC cohort. Patients who were under 50 years old were more likely to be female, have left-sided tumors, advanced stages (III/IV), aggressive histopathological features (e.g., signet ring cells and perineural invasion), BRAF mutations, a stronger family history of CRC, and a better ECOG performance status. Conversely, patients who were 50 years and older were predominantly male, with more right-sided tumors, earlier stages (I/II), and well-to-moderately differentiated tumors. Non-significant trends showed higher lymphovascular invasion and tumor obstruction in younger patients and a slightly higher BMI in older patients, indicating that younger patients with CRC may have more aggressive diseases possibly driven by unique etiological or biological factors. This necessitates further exploration of age-tailored management approaches. The survival analysis identified age 50 and above as an independent predictor of poorer survival. The forest plot further highlights that subgroups with tumor perforation, signet ring cell carcinoma, and stage II/III cancer exhibit even greater survival disadvantages in patients 50 years and older.

Overall, the survival of patients with EOCRC is influenced by a combination of demographic, clinical, and pathological factors, with the younger patients often presenting with a more advanced disease but potentially better survival outcomes when adjusted for stage and other factors. Further research is required to understand the underlying biological differences and improve the treatment strategies for this population.

In conclusion, although early-onset colon cancer presents with more aggressive characteristics, it is linked to improved survival compared with late-onset cases. Further research is necessary to confirm these observations.

## Supplementary Information

Below is the link to the electronic supplementary material.

Supplementary file1 (DOCX 156 KB)

## Data Availability

No datasets were generated or analysed during the current study.
