# Supplementary material for: Early- vs. late-onset colon cancer: clinicopathological insights and survival outcomes in an East Asian cohort
Source: Int J Colorectal Dis. 2025 Oct 9;40(1):212. doi: 10.1007/s00384-025-05007-4 (PMC12511223; doi:10.1007/s00384-025-05007-4)

Supplementary Table 1 Univariate and multivariate analyses for disease-free survival in patients with stage II/III colon cancer

| Variable | Univariate analysis | | |  | Multivariate analysis | | |  |
| --- | --- | --- | --- | --- | --- | --- | --- | --- |
|  | HR | 95% CI | *p* |  | HR | 95% CI | *p* |  |
| Age  ≧50  <50 | 1.827  - | 1.308–2.552  - | 0.001 |  | 1.908  - | 1.272–2.862  - | 0.002 |  |
| Sex  Male  Female | 1.225  - | 1.028–1.461  - | 0.024 |  | 1.164  - | 0.933–1.453  - | 0.179 |  |
| ECOG  0–1  2–4 | -  2.317 | -  1.762–3.047 | 0.001 |  | -  1.430 | -  0.968–2.113 | 0.072 |  |
| BMI  ≧25  <25 | 0.918  - | 0.765–1.101  - | 0.356 |  |  |  |  |  |
| Location  Right-sided  Left-sided | 1.020  - | 0.857–1.213  - | 0.824 |  |  |  |  |  |
| Tumor obstruction  Yes  No | 2.034  - | 1.690–2.448  - | 0.001 |  | 1.939  - | 1.547–2.431  - | 0.001 |  |
| Tumor perforation  Yes  No | 2.056  - | 1.352–3.127  - | 0.001 |  | 2.230  - | 1.262–3.942  - | 0.006 |  |
| Family history of CRC  Yes  No | 0.773  - | 0.598–0.999  - | 0.049 |  | 0.988  - | 0.715–1.366  - | 0.943 |  |
| Stage  II  III | 0.445  - | 0.372–0.532  - | 0.001 |  | 0.476  - | 0.359–0.630  - | 0.001 |  |
| Grade of differentiation  Well to moderate  Poor to undifferentiated | -  1.243 | -  0.91–1.699 | 0.172 |  | -  1.102 | -  0.764–1.588 | 0.603 |  |
| LVI  Presence  No | 2.220  - | 1.869–2.637  - | 0.001 |  | 1.829  - | 1.437–2.326  - | 0.001 |  |
| Presence of signet ring cells  Yes  No | 2.144  - | 1.541–2.982  - | 0.001 |  | 1.982  - | 1.316–2.984  - | 0.001 |  |
| Presence of PNI  Yes  No | 2.469  - | 2.004–3.041  - | 0.001 |  | 1.746  - | 1.366–2.232  - | 0.001 |  |
| CEA level (preOP)  ≧5 ng/mL  <5 ng/mL | 2.260  - | 1.887–2.708  - | 0.001 |  | 1.987  - | 1.595–2.474  - | 0.001 |  |
| NLR  ≧3  <3 | 1.506  - | 1.257–1.804  - | 0.001 |  | 1.051  - | 0.837–1.327  - | 0.656 |  |
| Oxaliplatin-based adjuvant chemotherapy  Yes  No | 1.188  - | 0.997–1.416  - | 0.054 |  | 0.622  - | 0.482–0.801  - | 0.001 |  |
|  |  |  |  |  |  |  |  |  |

Supplementary Table 2 Univariate and multivariate analyses for overall survival in the 3459 patients with colon cancer

| Variable | Univariate analysis | | |  | Multivariate analysis | | |  |
| --- | --- | --- | --- | --- | --- | --- | --- | --- |
|  | HR | 95% CI | *p* |  | HR | 95% CI | *p* |  |
| Age  ≧50  <50 | 1.627  - | 1.335–1.984  - | 0.001 |  | 1.897  - | 1.475–2.440  - | 0.001 |  |
| Sex  Male  Female | 1.343  - | 1.204–1.497  - | 0.001 |  | 0.804  - | 0.699–0.924  - | 0.002 |  |
| ECOG  0–1  2–4 | -  3.472 | -  2.942–4.098 | 0.001 |  | -  2.267 | -  1.774–2.898 | 0.001 |  |
| BMI  ≧25  <25 | 0.724  - | 0.645–0.813  - | 0.001 |  | 0.801  - | 0.691–0.928  - | 0.003 |  |
| Location  Right-sided  Left-sided | 1.191  - | 1.072–1.323  - | 0.001 |  | 1.330  - | 1.155–1.531  - | 0.001 |  |
| Tumor obstruction  Yes  No | 2.012  - | 1.786–2.267  - | 0.001 |  | 1.487  - | 1.283–1.725  - | 0.001 |  |
| Tumor perforation  Yes  No | 1.912  - | 1.420–2.576  - | 0.001 |  | 1.487  - | 1.283–1.725  - | 0.001 |  |
| Family history of CRC  Yes  No | 0.722  - | 0.617–0.844  - | 0.001 |  | 0.958  - | 0.788–1.165  - | 0.669 |  |
| Stage  I/II  III/IV | -  2.881 | -  2.577–3.221 | 0.001 |  | -  1.945 | -  1.641–2.305 | 0.001 |  |
| Grade of differentiation  Well to moderate  Poor to undifferentiated | -  1.672 | -  1.399–2.000 | 0.001 |  | -  1.357 | -  1.075–1.714 | 0.010 |  |
| LVI  Presence  No | 2.402  - | 2.161–2.670  - | 0.001 |  | 1.584  - | 1.356–1.850  - | 0.001 |  |
| Presence of signet ring cells  Yes  No | 1.623  - | 1.276–2.064  - | 0.001 |  | 1.139  - | 0.846–1.535  - | 0.390 |  |
| Presence of PNI  Yes  No | 2.481  - | 2.180–2.824  - | 0.001 |  | 1.643  - | 1.400–1.928  - | 0.001 |  |
| CEA level (preOP)  ≧5 ng/mL  <5 ng/mL | 2.890  - | 2.585–3.230  - | 0.001 |  | 2.116  - | 1.832–2.444  - | 0.001 |  |
| NLR  ≧3  <3 | 2.901  - | 2.873–2.335  - | 0.001 |  | 1.513  - | 1.312–1.746  - | 0.001 |  |
|  |  |  |  |  |  |  |  |  |

**Supplementary Fig. 1** Disease-free survival by age group in stage II/III colon cancer


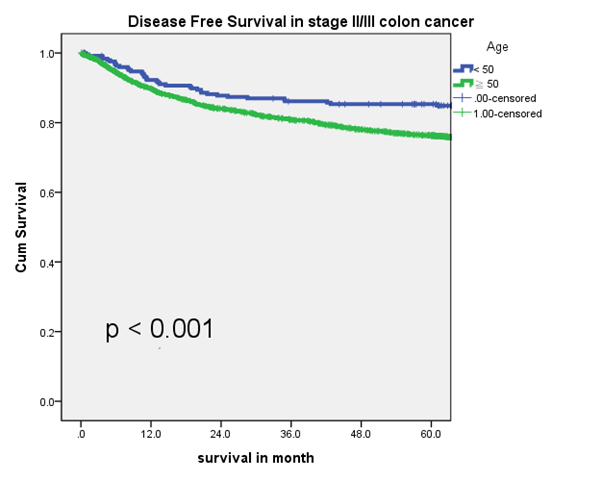


**Supplementary Fig. 2** Overall survival by age group in all patients with colon cancer


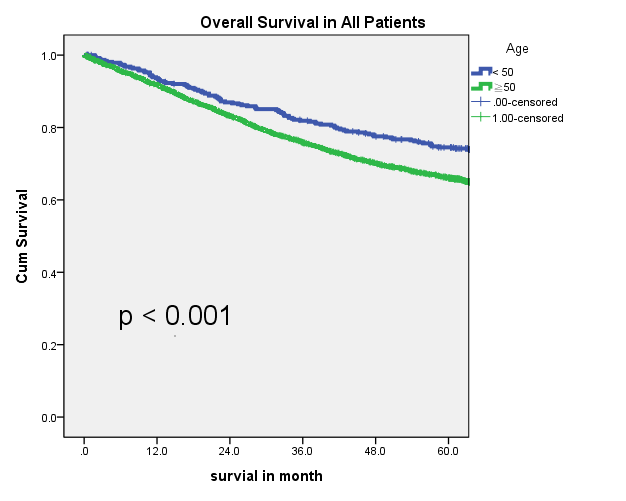


**Supplementary Fig. 3a** Age-stratified cancer-specific survival in stage I colon cancer (1494 patients lost to follow-up were excluded)


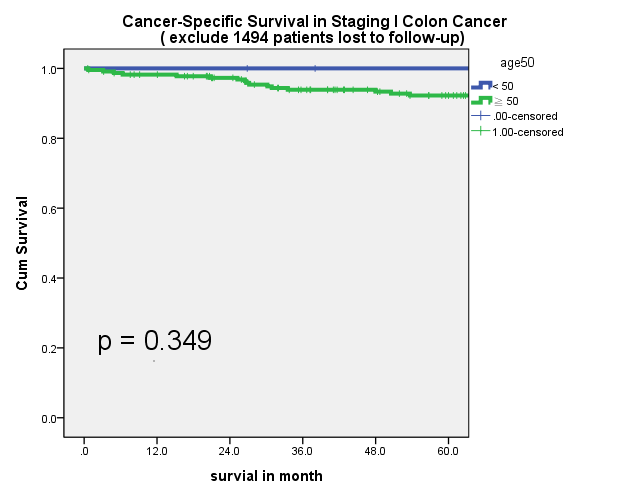


**Supplementary Fig. 3b** Age-stratified cancer-specific survival in stage II colon cancer (1494 patients lost to follow-up were excluded)


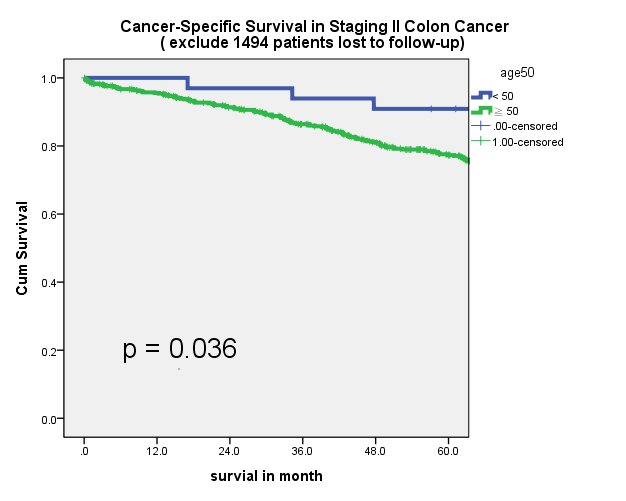


**Supplementary Fig. 3c** Age-stratified cancer-specific survival in stage III colon cancer (1494 patients lost to follow-up were excluded)


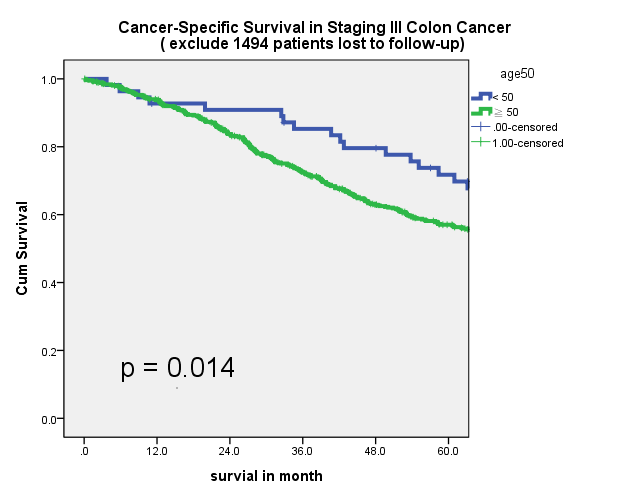


**Supplementary Fig. 3d** Age-stratified cancer-specific survival in stage IV colon cancer (1494 patients lost to follow-up were excluded)


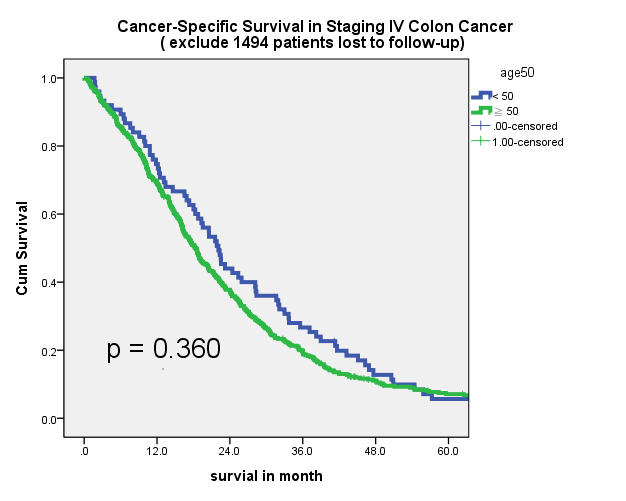

Supplement: Supplementary file 1 — Supplementary file1 (DOCX 156 KB) [file 384_2025_5007_MOESM1_ESM.docx]
